# Histone Methyltransferase Enhancer of Zeste Homolog 2-Mediated ABCA1 Promoter DNA Methylation Contributes to the Progression of Atherosclerosis

**DOI:** 10.1371/journal.pone.0157265

**Published:** 2016-06-13

**Authors:** Yun-Cheng Lv, Yan-Yan Tang, Ping Zhang, Wei Wan, Feng Yao, Ping-Ping He, Wei Xie, Zhong-Cheng Mo, Jin-Feng Shi, Jian-Feng Wu, Juan Peng, Dan Liu, Francisco S. Cayabyab, Xi-Long Zheng, Xiang-Yang Tang, Xin-Ping Ouyang, Chao-Ke Tang

**Affiliations:** 1 Institute of Cardiovascular Research, Key Laboratory for Atherosclerology of Hunan Province, Medical Research Center, Hunan Province Cooperative Innovation Center for Molecular Target New Drug Study, University of South China, Hengyang, Hunan, 421001, China; 2 Laboratory of Clinical Anatomy, University of South China, Hengyang, 421001, China; 3 The Key Laboratory of Carcinogenesis of the Chinese Ministry of Health and The Key Laboratory of Carcinogenesis and Cancer Invasion of the Chinese Ministry of Education, Cancer Research Institute, Central South University, Changsha, Hunan, 410013, China; 4 School of Electronics and Information Engineering, Hunan University of Science and Engineering, Yongzhou, Hunan, 425100, China; 5 School of Information Science and Engineering, Central South University, Changsha, Hunan, 410000, China; 6 Department of Surgery, College of Medicine, University of Saskatchewan, Saskatoon, Saskatchewan, S7N 5E5, Canada; 7 Department of Biochemistry and Molecular Biology, The Libin Cardiovascular Institute of Alberta, The University of Calgary, Health Sciences Center, 3330 Hospital Dr NW, Calgary, Alberta, T2N 4N1, Canada; 8 Department of Radiology and Imaging Sciences, Emory University School of Medicine, WCI Suite C5018, 1701 Uppergate Drive, Atlanta, GA, 30322, United States of America; Beijing Key Laboratory of Diabetes Prevention and Research, CHINA

## Abstract

ATP-binding cassette transporter A1 (ABCA1) plays a critical role in maintaining cellular cholesterol homeostasis. The purpose of this study is to identify the molecular mechanism(s) underlying ABCA1 epigenetic modification and determine its potential impact on ABCA1 expression in macrophage-derived foam cell formation and atherosclerosis development. DNA methylation induced foam cell formation from macrophages and promoted atherosclerosis in apolipoprotein E-deficient (apoE^−/−^) mice. Bioinformatics analyses revealed a large CpG island (CGI) located in the promoter region of ABCA1. Histone methyltransferase enhancer of zeste homolog 2 (EZH2) downregulated ABCA1 mRNA and protein expression in THP-1 and RAW264.7 macrophage-derived foam cells. Pharmacological inhibition of DNA methyltransferase 1 (DNMT1) with 5-Aza-dC or knockdown of DNMT1 prevented the downregulation of macrophage ABCA1 expression, suggesting a role of DNA methylation in ABCA1 expression. Polycomb protein EZH2 induced DNMT1 expression and methyl-CpG-binding protein-2 (MeCP2) recruitment, and stimulated the binding of DNMT1 and MeCP2 to ABCA1 promoter, thereby promoting ABCA1 gene DNA methylation and atherosclerosis. Knockdown of DNMT1 inhibited EZH2-induced downregulation of ABCA1 in macrophages. Conversely, EZH2 overexpression stimulated DNMT1-induced ABCA1 gene promoter methylation and atherosclerosis. EZH2-induced downregulation of ABCA1 gene expression promotes foam cell formation and the development of atherosclerosis by DNA methylation of ABCA1 gene promoter.

## Introduction

It is well known that monocyte-derived macrophages are the main factors in the development of atherosclerosis, and ATP-binding cassette transporter A1 (ABCA1) is highly expressed in macrophages [1, 2]. The function of ABCA1 is to mediate apolipoprotein A-I (apoA-I)-dependent cholesterol efflux, which has been identified as an important target in the treatment of atherosclerosis [1, 3]. ABCA1 expression in macrophages is regulated by the liver X receptor agonists, and also inhibited by microRNAs. ABCA1 upregulation inhibits the formation of foam cells. Mice lacking ABCA1 are prone to atherosclerosis when challenged with a high-fat diet [4, 5]_,_ suggesting a critical role for ABCA1 in atheroprotection.

Transcription of ABCA1 is largely controlled by its proximal promoter, which is highly conserved between mouse and human [6]. Although little is known about epigenetic modulation of ABCA1 expression, emerging evidence suggests the roles for the histone modifier, histone methyltransferase enhancer of zeste homolog 2 (EZH2), that is involved in DNA methyltransferase 1 (DNMT1) recruitment [7]. It was our hypothesis that EZH2 may regulate ABCA1 gene expression by regulating DNMT1 recruitment.

ABCA1, atherosclerosis and endothelial cell homeostasis are associated with epigenetic changes *in vitro* and *in vivo* as described in experimental animal models and humans [8–10]. Apolipoprotein E-deficient (apoE^−/−^) mice fed a high-fat diet have aberrant DNA methylation patterns, including decreased global methylation in aorta and peripheral blood mononuclear cells, and special gene hypermethylation in atherosclerotic tissue samples [11]. The matrix metalloproteinase-2 (MMP-2) and MMP-9 genes are two genes epigenetically modified by oxidized low-density lipoprotein (ox-LDL) [12]. Both MMP-2 and MMP-9 are involved in extracellular matrix remodeling, and have been implicated in increased susceptibility to cardiovascular diseases [12, 13]. To date, evidence has shown that MMP-2 and MMP-9 are the only two genes which epigenetic modification may contribute to the development of cardiovascular disease. Considering the possibility that macrophage ABCA1 might also be epigenetically regulated by EZH2 in the hypercholesterolemic environment, we investigated whether EZH2 altered ABCA1 gene expression, and explored the roles of epigenetic DNA regulation in the development of atherosclerosis in apoE^−/−^ mice.

## Materials and Methods

### Cell culture

THP-1 cells were obtained from American type culture collection (ATCC, Rockefeller, USA), and RAW264.7 macrophages from the Institute of Life Science Research Center, Shanghai (Chinese Academy of Science, Shanghai, China). Both cell lines were maintained in RPMI Medium 1640 supplemented with 10% fetal bovine serum (FBS) at 37°C in an atmosphere containing 5% CO_2_. After 3‒5 days, THP-1 cells were treated with phorbol-12-myristate-13-acetate (PMA, 160 nmol/L; Sigma, St. Louis, USA) for 48 h. The medium was then replaced with a serum-free medium containing ox-LDL (50 μg/ml) for 48 h to transform THP-1 cells to foam cells. For the experiments, cells were infected with control lentivirus (LV-mock, MCS-3FLAG-SV40-puromycin), recombinant lentivirus (LV-EZH2, Ubi- MCS-3FLAG-SV40-puromycin as the vector), and DNMT1 shRNA (human shDNMT1: 5’-GATCCCCCACTGGTTCTGCGCTGGGATTCAAGAGATCCCAGC GCAGAACCAGTGTTTTTGGAAA-3’, mouse shDNMT1: 5’-GATCCCCGAACGG CATCAAGGTGAACTTCAAGAGAGTTCACCTTGATGCCGTTCTTTTTGGAAA-3’, hU6-MCS-CMV-Puromycin as the vector, GeneChem, Shanghai, China) using Polyfect-V-v infection reagent according to manufacturer’s instructions.

### Animals protocol

Six-week-old male apoE^−/−^ mice (n = 47; C57BL/6) were purchased from Department of Laboratory Animal Science of Peking University (Beijing, China). After fed with a rodent chow diet containing 4.5% (w/w) fat for a week, apoE^−/−^ mice were randomly divided into three groups: control group (n = 9); high fat diet (HF) group (n = 19); high fat diet and EZH2 overexpression (HF+EZH2) group (n = 19). Control group was still fed on rodent chow diet. The other two groups were switched to and maintained on Western diet (WD) containing 21% (w/w) fat and 1.25% (w/w) cholesterol for 12 wk. To study the role of EZH2-induced ABCA1 methylation in atherosclerosis, recombinant lentiviruses encoding mouse EZH2 (LV-EZH2) were commercially generated by packing EZH2 cDNA (NM_007971) sequence into Ubi- MCS-3FLAG-SV40-puromycin vector (GeneChem, Shanghai, China). At the end of 8 wk, mice in control and HF groups were injected via the tail vein with 1 × 10^7^ transducing units (TU)/mouse control lentivirus (LV-mock) and with the isodose of LV-EZH2 in HF+EZH2 group, respectively [14]. After fasting for 6 h at the end of 12 wks, mice were euthanized, followed by blood-sampling from the retro-orbital plexus and collected tissues including aorta, heart, liver and kidney for further analyses. All experimental procedures conformed to the Guide for the Care and Use of Laboratory Animals published by the US National Institutes of Health (NIH Publication No. 85–23, revised 1996) and was approved by the Institutional Animal Ethics Committee of University of South China.

### Histological analysis

Atherosclerotic lesions were quantified by en face analysis of the whole aorta and by cross-sectional analysis of the proximal aorta. For en face analysis of aortas, aortas were photographed with stereomicroscope (Nikon SMZ-1500, Tokyo, Japan) after the careful removal of adventitial tissue, then opened longitudinally, stained with Sudan IV (Sigma, St. Louis, America), and pinned on a white wax surface. The Sudan IV-stained aortas were photographed (Nikon Coolpix 990, Japan) for the quantification of atherosclerotic lesions. The total aortic surface area and the lesion area were measured by image analysis (ImageJ software 7.0, NIH, America), and the ratio of the lesion area to the total area was calculated.

For the cross-sectional analysis of aortas, the upper portions of the hearts and proximal aortas were obtained, embedded in Optimal Cutting Temperature (OCT) compound (Fisher, America), and stored at -20°C. Serial 8-μm thick cryosections of the aortas, beginning at the aortic sinus, were collected for a distance of 240 μm. Ten sections per animal were stained for atherosclerotic lesions with hematoxylin and eosin staining (HE), lipids content with Oil-red O (Sigma, St. Louis, America) and collagen content with Masson's trichrome (MT) staining (Senbeibio, Nanjing, China). Lesion areas and collagen contents were quantified using Image-Pro Plus 6.0 software (Media Cybernetics, America). Data were expressed as lesion size ± SEM.

### Databases and bioinformatics analyses

The ABCA1 promoter sequences were obtained from the NCBI Sequence database. The presence of CpG islands within ABCA1 promoter was predicted by EMBOSS CpGplot program (http://www.ebi.ac.uk/emboss/cpgplot/) and Cpgislands (http://cpgislands.usc.edu/). Transcription factor binding sites were predicted by JASPAR (http://jaspar.genereg.net/). The conservation of ABCA1 CpG islands among different species was examined using the BLAST of NCBI (http://blast.ncbi.nlm.nih.gov/Blast.cgi?CMD=Web&PAGE_TYPE=BlastHome).

### Oil Red O stain

To quantify cellular lipid accumulation, the foam cells were stained with Oil Red O. THP-1 cells were treated with 160 nM PMA for 48 h. The medium was then replaced with a serum-free medium containing 50 μg/ml ox-LDL and 25 μg/ml human apoA-I for 48 h. After the fully differentiate THP-1/RAW264.7 cells to foam cells, the foam cells were verified by fixing cells with 4% paraformaldehyde and subsequently staining with 0.5% Oil Red O. Hematoxylin was used for counterstaining cell nuclei, and cells were photographed at ×400 magnification.

### Cholesterol efflux assay

Cells were cultured at 60% confluence, and then labeled with 0.2 mCi/ml [^3^H] cholesterol for 24 h. After 24 h, cells were washed and equilibrated with fresh media. Equilibrated [^3^H] cholesterol-labeled cells were then washed with PBS and incubated in efflux medium containing RPMI 1640 medium and 0.1% BSA with 25 μg/ml human plasma apoA-I for 6 h. The medium was removed and centrifuged at 14,000 g for 10 min. Total cell-associated radioactivity was determined by dissolving the cells in isopropanol. Medium and cell-associated [^3^H] cholesterol was then measured by liquid scintillation counting. The percent efflux was calculated by the following equation: [total media count/(total cellular count + total media count)]×100%.

### Western blot and real-time PCR analyses

Western blot and real-time PCR were applied to examine the levels of protein and mRNA, respectively. Tissues and cells were lysed for protein extraction and separated by SDS-PAGE. ABCA1 antibody was purchased from Abcam (Cambridge, UK). We determined the levels of β-actin using anti-mouse-β-actin antibody as a loading control (Beyotime, Shanghai, China). In general, the membranes were washed three times in Tris-buffered saline containing 0.1% Tween (TBST) and then incubated for 2 h with peroxidase-conjugated secondary antibodies. After three washes of 15 min each with TBST, the proteins were visualized using chemiluminescence. Total RNA was extracted using TRIzol reagent (Invitrogen, Carlsbad, USA) in accordance with the manufacturer’s instructions. Sequences of primers were described in our previous publication [15]. Quantitative measurements were determined using the Ct method, and GAPDH was used as the internal control.

### Methylation-specific PCR

DNA was extracted from THP-1 and RAW264.7 cells and mouse aortas by the DNA extraction kit (Qiagen China, Shanghai, China), and stored at -20°C. Bisulfite modification of DNA was performed with the EZ DNA Methylation-Gold kit (Zymo research, Orange, USA) according to the manufacturer’s instructions. Methylation-specific PCR was then carried out to determine the methylation status of ABCA1. Bisulphite-modified DNA was used for PCR with primers specifically designed for methylated or unmethylated sequences. PCR reactions (40 cycles) were performed using denaturation at 95°C for 30 s, annealing at 56°C for 30 s, and elongation at 72°C for 30 s. Products were separated by gel electrophoresis. Methylation levels (%) were calculated by the density of the methylated band vs. total density of the unmethylated (UM) and methylated (M) bands.

### Chromatin immunoprecipitation (ChIP) assay

ChIP assays were performed with a ChIP Assay Kit (Millipore, Bedford, USA) with the indicated antibodies. Briefly, chromatin associated with specific immunoprecipitation or non-immune mouse IgG control was used as a template for PCR amplification of a specific ABCA1 promoter sequence. Primers (forward 5'- AGGAGCCCGAGTAAATTG-3', reverse 5'-CGGCAACAAGCAGAAGAA-3') were designed to target a 162 bp fragment of ABCA1 promoter. PCR amplification for glyceraldehydes-3-phosphate dehydrogenase (GAPDH) was used as a negative control (GAPDH primers: forward 5'-GAGCTGAACGGGAAACTCAC-3', reverse 5'-GGTCTGGGATGGAAACTGTG-3'; a 146 bp fragment).

### Statistical analysis

Data were expressed as the mean ± standard deviation (S.D.) of at least three independent experiments. Data were analyzed by one-way ANOVA with Graphpad Prism 5 software. Values with *P*<0.05 were considered as statistical significance.

## Results

### Overexpression of EZH2 induces lipid accumulation in macrophages and accelerates the progression of atherosclerotic lesions in apoE^−/−^ mice

**Fig 1:**
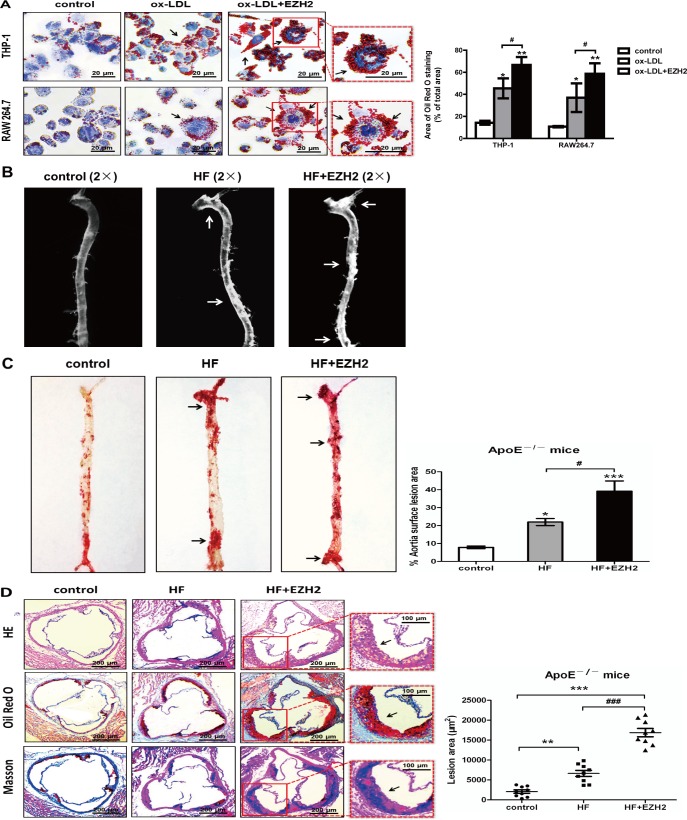
Effects of EZH2 on macrophage cholesterol accumulation and aortic plaque. A, EZH2 enhanced cholesterol accumulation in THP-1 and RAW264.7 macrophage-derived foam cells. THP-1/RAW264.7 macrophages were stained for lipids with Oil red O. The nuclei were stained with hematoxylin. Control: 25 μg/ml human apoA-I alone but infected with LV-mock vector; ox-LDL: 25 μg/ml human apoA-I, 50 μg/ml ox-LDL with LV-mock vector; ox-LDL+EZH2: 25 μg/ml human apoA-I, ox-LDL (50 μg/ml) with LV-EZH2 vector. The arrow indicates foam cells visualized under a light microscope with ×400 magnification, scale bar, 20 μm. Results are representative of 3 independent experiments. Bar graph data is based on Image-Pro Plus software. Mean ± S.D., one-way ANOVA, *: *P*<0.05 and **: *P*<0.01 *vs*. control, #: *P*<0.05 *vs*. ox-LDL+EZH2. B, Stereomicrographs showing atherosclerotic plaques of apoE^−/−^ mice (arrows). Control group: apoE^−/−^ mice fed on chow diet and injected with LV-mock; HF group: apoE^−/−^ mice fed on high fat diet and injected with LV-mock; HF+EZH2 group: apoE^−/−^ mice fed on high fat diet and injected with LV-EZH2. C, The plaque formation showed by Sudan IV staining. The area of the lesion in % of the total aortic area, n = 9, mean ± SEM, one-way ANOVA, *: *P*<0.05 and ***: *P*<0.001 *vs*. control, #: *P*<0.05 *vs*. HF+EZH2. D, Photomicrographs showing aortic sinus lesions of apoE^−/−^ mice. The atherosclerotic plaque is stained with HE, Oil red O and MT. Scale bar, 200 μm in left panel, 100 μm in right panel. The area of aortic sinus lesion, n = 9, each group. Mean ± SEM, **: *P*<0.01 and ***: *P*<0.001 *vs*. control, ###: *P*<0.001 *vs*. HF+EZH2.

To investigate the effects of EZH2 on lipid accumulation during foam cell formation, we performed lipid analyses of THP-1 and RAW264.7 cells with Oil Red O staining. The results showed that cells in the control without ox-LDL incubation did not contain high levels of lipid droplets (Fig 1A, left panels). Treatment of macrophages with 50 μg/mL ox-LDL for 48 h resulted in foam cell formation which was characterized by heavy lipid loading (Fig 1A, middle panels). Overexpression of EZH2 markedly exacerbated cellular lipid accumulation (Fig 1A, right panels) in both THP-1- and RAW264.7-derived foam cells. Similarly, the area of lipid droplets in EZH2 overexpression group was significantly higher than the area in control and ox-LDL (Fig 1A).

After above *in vitro* experiments, the effects of EZH2 on the atherosclerosis were examined in six-week-old male apoE^−/−^ mice. Our results showed that lentivirus-mediated overexpression of EZH2 resulted in an increase in atherosclerotic lesions as assessed by stereomicroscopy and Sudan IV staining (Fig 1B and 1C). The development of atherosclerotic lesions in the aortic sinus was also examined using HE staining. As shown in Fig 1D (left panel), LV-EZH2 infection markedly increased atherosclerotic plaque sizes, compared with those in the control group. In addition, the effects of EZH2 on lipid and collagen contents in the atherosclerotic plaques of the aortic sinus were determined using Oil Red O and MT staining. Data in the Fig 1D (middle and right panels) showed that the lipids and collagen areas in atherosclerotic plaques of LV-EZH2-infected mice were markedly increased compared with those in LV-mock-infected apoE^−/−^ mice. Consistent with the statistic data, atherosclerotic areas of aortic sinus increased upon treatment with LV-EZH2 (Fig 1D).

### EZH2 inhibits cellular cholesterol efflux and ABCA1 expression

**Fig 2:**
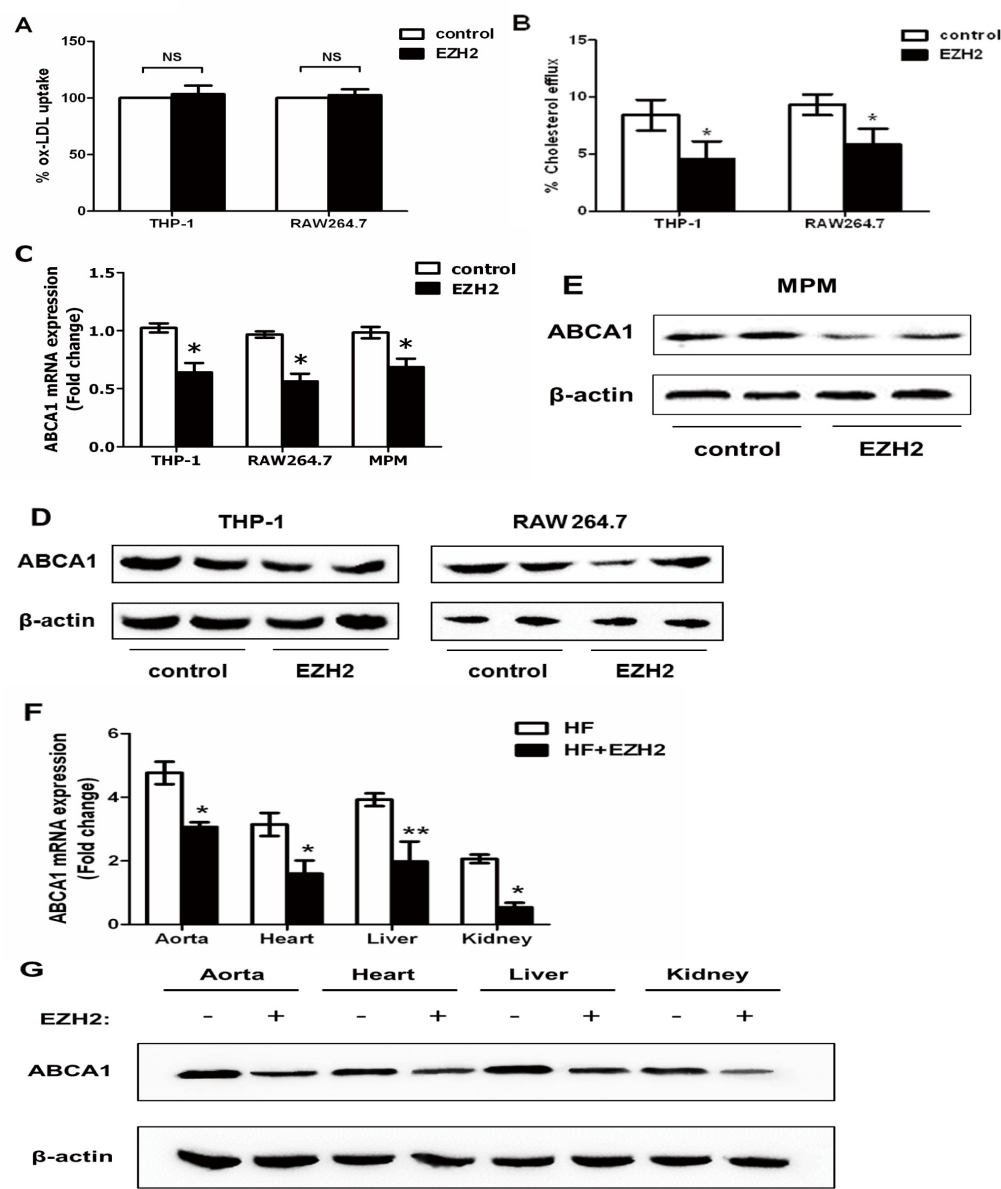
Effects of EZH2 on cholesterol efflux and ABCA1 expression. A, DiI-oxLDL (1 μg/ml) was added to THP-1/RAW264.7 macrophages infected with LV-mock or LV-EZH2 vectors. After incubation for 24 h, the uptake was analyzed by flow cytometry. The control values were set to 100%, and then uptake data were calculated as the percentage of ox-LDL uptake *vs*. the control. B, Cholesterol efflux assay was performed using liquid scintillation counting assays as described in Methods. Cholesterol efflux of RAW264.7 macrophages were preloaded with [^3^H] cholesterol-oxLDL. C, EZH2 down-regulated ABCA1 mRNA in THP-1/RAW264.7 macrophages and mouse peritoneal macrophages (MPM). ABCA1 mRNA was quantified in THP-1 and RAW264.7 infected with LV-mock or LV-EZH2 vectors for 24 h or in MPM harvested from the peritoneal cavity of apoE^−/−^ mice 3 d after intraperitoneal injection with 2 ml thioglycollate. D, EZH2 down-regulated ABCA1 protein in both THP-1 and RAW264.7 macrophages. E, EZH2 downregulated ABCA1 protein in MPM harvested from the peritoneal cavity of apoE^−/−^ mice 3 d after intraperitoneal injection with 2 ml thioglycollate. Representative immunoblots show the effect of EZH2 on ABCA1 expression. Mean ± S.D., one-way ANOVA, *: *P*<0.05 and **: *P*<0.01 *vs*. control. F, EZH2 down-regulated ABCA1 mRNA in aorta, heart, liver and kidney of apoE^−/−^ mice. RNA from the indicated organs was isolated from apoE^−/−^ mice injected with LV-mock (n = 5, solid bars) or LV- EZH2 (n = 5, open bars). Mean ± S.D., one-way ANOVA, *: *P*<0.05 *vs*. HF+mock group. G, EZH2 downregulated ABCA1 protein in aorta, heart, liver and kidney of apoE^−/−^ mice. Representative immunoblots show the effect of EZH2 on ABCA1 expression. Proteins from the indicated organs were isolated from apoE^−/−^ mice treated with LV-mock or LV-EZH2 (n = 5).

Next, we examined whether EZH2 affected lipid accumulation through the regulation of ox-LDL uptake and cholesterol efflux. THP-1-derived macrophages were incubated with fluorescence-labeled ox-LDL (DiI-ox-LDL) for 24 h, followed by the analyses using flow cytometry. There were no significant changes in the ox-LDL uptake rates in macrophages infected with control and LV-EZH2 vector (Fig 2A), suggesting that EZH2 had no or very little, if any, effect on lipid uptake of macrophages. Our results showed that EZH2 infection significantly inhibited cholesterol efflux from THP-1 and RAW264.7 macrophage-derived foam cells (Fig 2B), suggesting that EZH2 promotes lipid accumulation in foam cells by inhibiting cholesterol efflux.

Therefore, we further investigated whether EZH2 affected the expression of ABCA1, the primary transporter responsible for cholesterol efflux from THP-1 and RAW264.7 macrophage-derived foam cells [16, 17]. Our results showed that EZH2 did suppress ABCA1 expression at both mRNA and protein level in THP-1 and RAW264.7-derived foam cells (Fig 2C and 2D, S1A Fig). We also observed that EZH2 infection reduced ABCA1 mRNA and protein levels in mouse peritoneal macrophages (MPM), aortas, hearts, livers and kidneys of apoE^−/−^ mice (Fig 2E, 2F and 2G, S1B Fig). Taken together, our data suggest that EZH2 is involved in the regulation of lipid accumulation and atherosclerosis development by inhibiting ABCA1-mediated cholesterol efflux.

### EZH2-mediated DNA methylation of ABCA1 promoter

**Fig 3:**
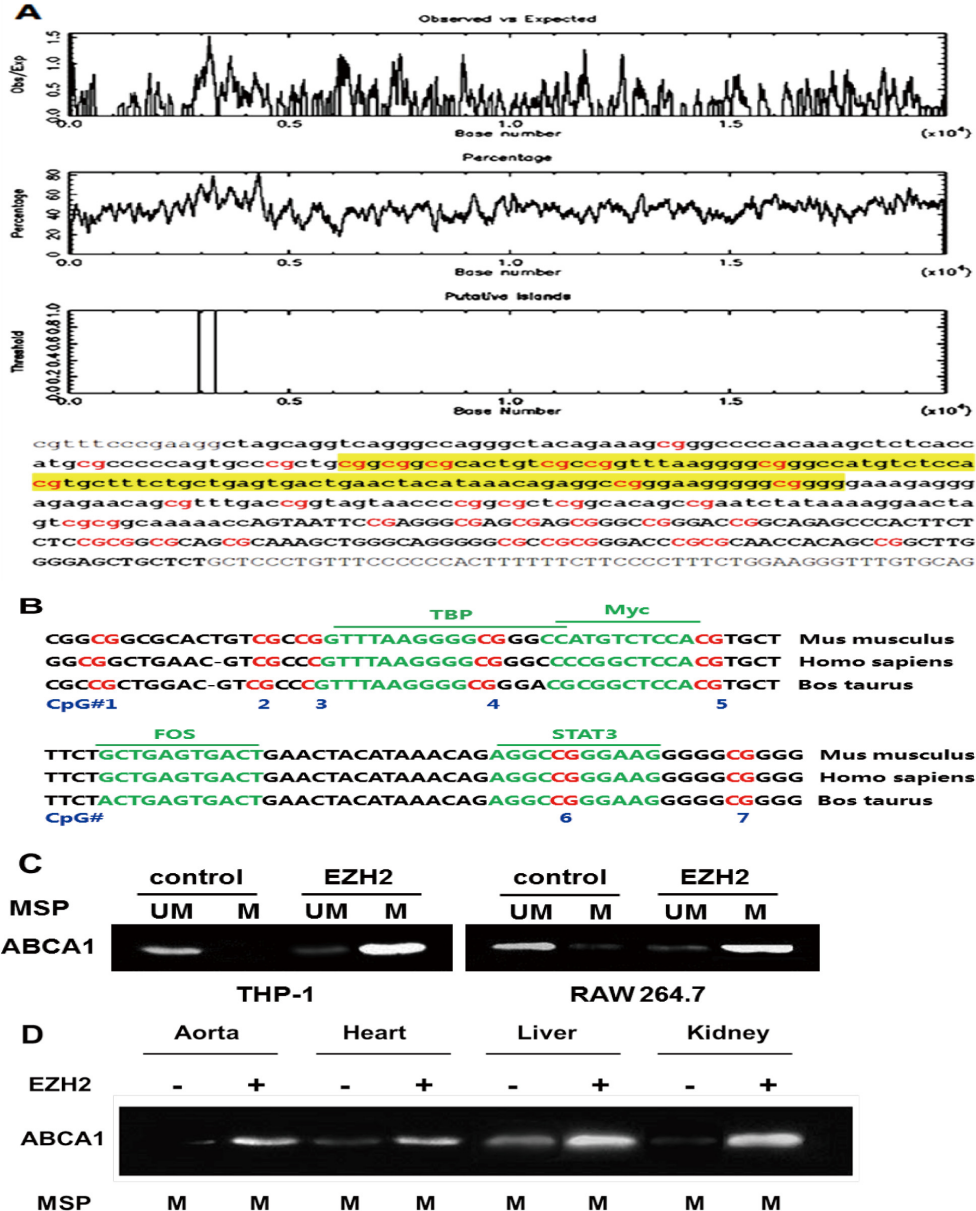
CpG islands identified in ABCA1 promoter sequence are well conserved in evolution and ABCA1 hypermethylation occurs in plaque-laden aorta. A, Observed/Predicted ratio of presence of CpG islands in ABCA1 promoter sequence (highlighted in yellow). Genomic position of promoter and ABCA1 CGIs obtained from different online websites (http://www.ebi.ac.uk/Tools/emboss/cpgplot/ and http://cpgislands.usc.edu/). B, Sequence alignment showing well conserved CpG islands and the binding sites of transcription factors. The conservation of ABCA1 CGI in some mammals obtained from the BLAST of NCBI (http://blast.ncbi.nlm.nih.gov/Blast.cgi?CMD=Web&PAGE_TYPE=BlastHome), and the transcription factor binding site is predicted by JASPAR (http://jaspar.genereg.net/). C, Hypermethylation of ABCA1 promoter in THP-1 and RAW264.7 macrophage-derived foam cells after EZH2 infection. PCR primers specific to unmethylated and methylated bisulfite-modified DNA were used to amplify the promoter of ABCA1 gene after EZH2 treatment. D, ABCA1 promoter methylation of the apoE^−/−^ mouse aorta, heart, liver and kidney was analyzed by MSP. UM: unmethylated; M: methylated.

We used the UCSC Human Genome Browser and NCBI gene bank (http://www.ncbi.nlm.nih.gov/pubmed/) to define the genomic features of the ABCA1 promoter sequence. As expected, ABCA1 promoter contained a single well-defined genic CGI as analyzed with the tools in the online website (http://www.ebi.ac.uk/Tools/emboss/cpgplot/) (Fig 3A). The ABCA1 CGI extended across 389 bp, containing a CG content of 57.4% with an observed-to-expected CpG ratio of 0.664, indicating a well-defined CGI compared with the control standard (Table 1). We also found that this CGI was conserved in some mammals, including mice and cows (Fig 3B). Information from the online website JASPAR indicated the presence of highly conservative putative binding sites for transcription factors, such as TBP, Myc, FOX and STAT3 with the CGI. Detailed sequence analysis suggests the presence of a core region that covers the center section with a higher CpG load. Taken together, these bioinformatics analyses suggest that ABCA1 promoter has the high probability to be modified by DNA methylation.

**Table 1:** Comparison of the human ABCA1 CGI to standard and stringent CGI definitions.

Criteria	CGI definition Standard	Stringent	ABCA1 CGI
Size (bp)	≥200	≥500	389
% C or G	>50	>55	57.4
CpG ratio*	>0.6	>0.65	0.66

*: Ratio of observed to expected number of CpGs.

In order to determine whether EZH2 could downregulate ABCA1 expression via DNA methylation, we examined DNA methylation of ABCA1 promoter by methylation-specific PCR. In THP-1 and RAW264.7 macrophages, EZH2 increased ABCA1 promoter DNA methylation (Fig 3C), suggesting a role for DNA methylation in the effects of EZH2 on ABCA1 expression. In our *in vivo* study, EZH2 promoted the development of atherosclerosis in aortas of apoE^−/−^ mice, which was associated with the higher DNA methylation level on ABCA1 promoter (Fig 3D). Thus, DNA methylation might be crucial in silencing ABCA1 expression *in vitro* and *in vivo*, likely contributing to its acceleration of atherogenesis.

### DNMT1 and MeCP2 are recruited to ABCA1 promoter by EZH2

**Fig 4:**
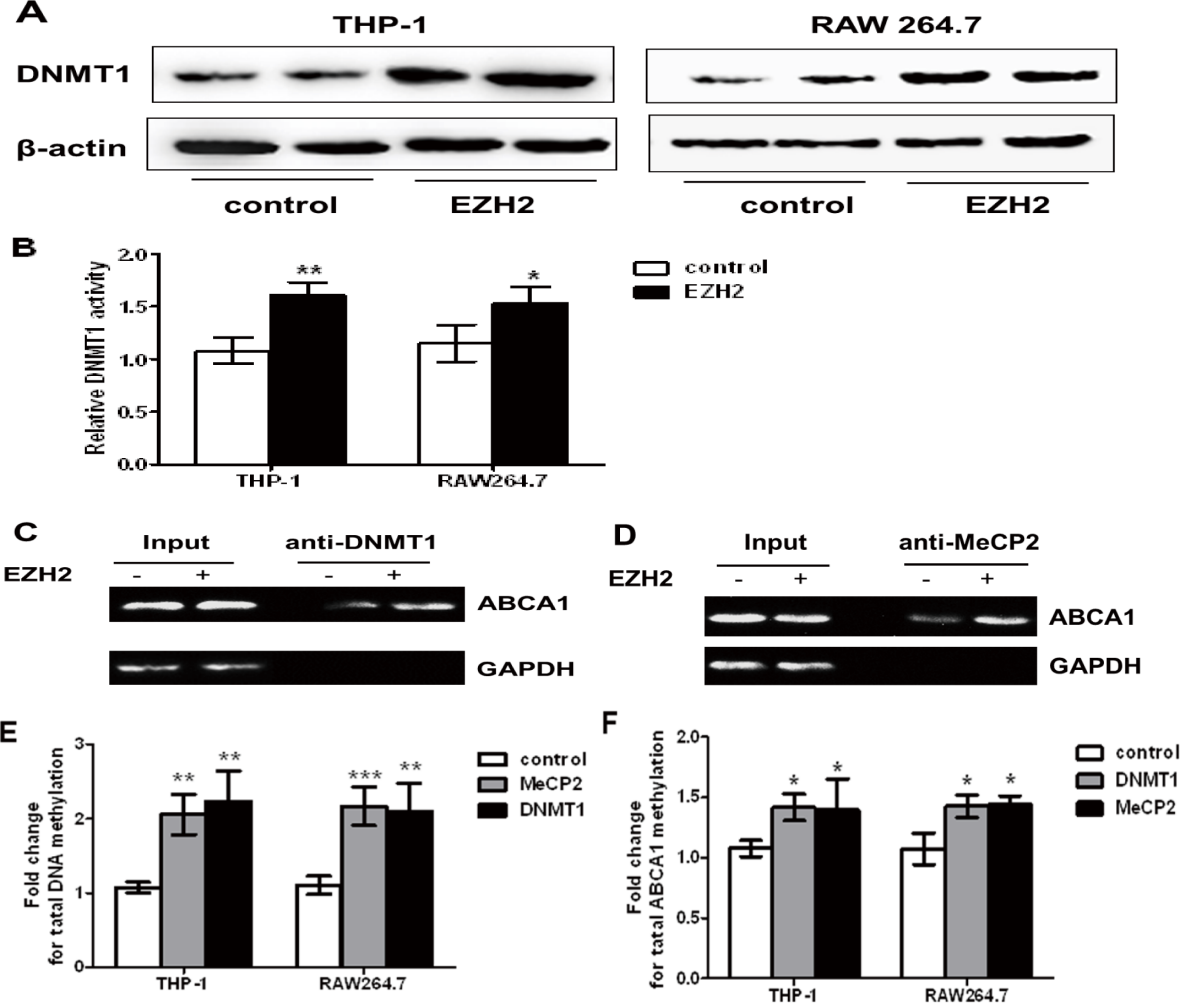
The effects of EZH2 on DNMT1/MeCP2 expression and recruitment. A, EZH2 upregulated DNMT1 expression. DNMT1 protein was quantified in THP-1 and RAW264.7 macrophages infected with LV-EZH2 vector for 24 h, normalized to β-actin, and is expressed as fold change compared with LV-mock vector-infected cells. B, EZH2 upregulated DNMT1 activity. DNMT1 activity was quantified in RAW264.7 macrophages infected with LV-EZH2 vector for 24 h and compared with LV-mock vector-infected cells. EZH2 increased the binding of DNMT1 and MeCP2 to the ABCA1 promoter. C and D, ChIP results of DNMT1 and MeCP2 in RAW 264.7 cells with LV-mock or LV-EZH2 vector for 24 h. RAW 264.7 cells were infected with LV-DNMT1 and LV-MeCP2. Unprecipitated chromatin was used as input. E, Genomic DNA methylation after DNMT1 and MeCP2 overexpression. F, Bisulfite promoter analysis for ABCA1-methylation after DNMT1 and MeCP2 overexpression. Mean ± S.D., one-way ANOVA, *: *P*<0.05, **: *P*<0.01 and ***: *P*<0.001 *vs*. control.

In order to further study the epigenetic mechanism underlying ABCA1 silencing, we determined whether EZH2 up-regulated the expression of DNMT1 and subsequently promoted the recruitment of DNMT1 to the ABCA1 promoter. As expected, our results showed that EZH2 significantly increased DNMT1 expression and total DNMT activity in macrophages (Fig 4A and 4B). Furthermore, the ChIP assays showed that EZH2 promoted the binding of DNMT1 to ABCA1 promoter (Fig 4C), suggesting that EZH2 up-regulates the expression of DNMT1 and enhances recruitment and binding of DNMT1 to the proximal region of ABCA1 promoter in macrophages.

Methyl-CpG-binding protein-2 (MeCP2) binds to methylated CpG dinucleotides that are often in close proximity to an AT-rich DNA sequence. A putative MeCP2-binding site exists within the proximal region of ABCA1 promoter. To test whether EZH2 stimulates binding of MeCP2 to its putative binding site in ABCA1 promoter, we performed the ChIP assays, and revealed that EZH2 indeed promoted the binding of MeCP2 to ABCA1 promoter (Fig 4D). DNMT1 and MeCP2 overexpression in THP-1 and RAW264.7 cells resulted in higher DNA methylation when compared with control (Fig 4E, S2 and S3 Figs). Bisulfite conversion experiments revealed that the methylation status of the ABCA1 promoter was increased in response to DNMT1 and MeCP2 overexpression (Fig 4F). Collectively, these data indicated that EZH2-promoted methylation of ABCA1 promoter regulates the recruitment of DNMT1 and MeCP2 to ABCA1 gene promoter.

### Inhibition of DNMT1 reverses the effect of EZH2

**Fig 5:**
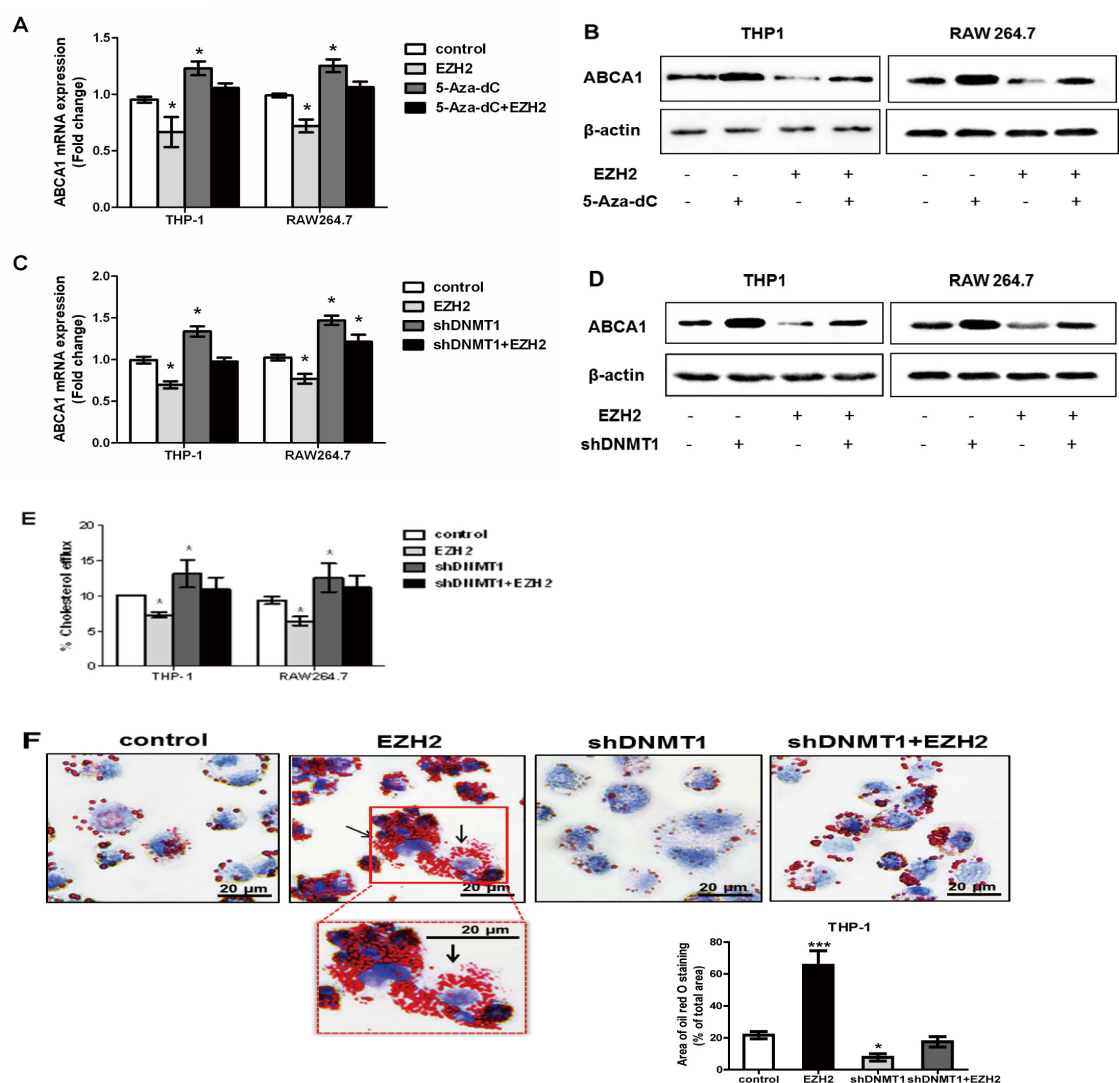
EZH2 regulates macrophage ABCA1 expression and cholesterol via DNA methylation. A and B, Inhibition of DNMT1 with 5-Aza-dC prevented EZH2-induced down-regulation of ABCA1 mRNA and protein. ABCA1 mRNA and protein were quantified and normalized to β-actin in THP-1 and RAW264.7 macrophages infected with LV-EZH2 vector for 24 h and with or without 5-Aza-dC treatment. C and D, Knockdown of DNMT1 with small hairpin RNA (shDNMT1) blocked EZH2-induced suppression of ABCA1 mRNA and protein. ABCA1 mRNA and protein were quantified and normalized to β-actin in THP-1 and RAW264.7 macrophages infected with LV-EZH2 vector for 24 h in the presence or absence of shDNMT1. E, Knockdown of DNMT1 through infection with shDNMT1 confronted EZH2-inhibited cholesterol efflux in THP-1 and RAW264.7 macrophages. Cholesterol efflux assay was performed using liquid scintillation counting assays as described in Methods. Cholesterol efflux of THP-1 and RAW264.7 macrophage were preloaded with [^3^H] cholesterol-oxLDL previously. F, Knockdown of DNMT1 through infection with shDNMT1 abolished EZH2-promoted cholesterol accumulation in THP-1 foam cells. THP-1 macrophage-derived foam cells were stained with Oil Red O. The nuclei were counter-stained with hematoxylin. The magnification of each panel is ×400. Mean ± S.D., one-way ANOVA, *: *P*<0.05 and **: *P*<0.01 *vs*. control.

The experiments described above have demonstrated the detrimental effects of EZH2 on ABCA1-mediated cholesterol efflux from THP-1 and RAW264.7 macrophage-derived foam cells (Fig 2B). Next, we used 5-Aza-dC to inhibit DNMT activity and then examine the role of DNA methylation in EZH2 effects on ABCA1-mediated cholesterol efflux. Our results showed that EZH2 had no inhibitory effect on ABCA1 expression at either mRNA or protein levels in the presence of 5-Aza-dC (Fig 5A and 5B). Similarly, shRNA knockdown of DNMT1 abolished EZH2-induced downregulation of ABCA1 mRNA and protein expression (Fig 5C and 5D, S2 Fig).

We also determined whether the inhibition of DNMT1 with small hairpin RNA (shDNMT1) altered ABCA1-mediated cholesterol efflux in THP-1 and RAW264.7 macrophage-derived foam cells. Data in Fig 5E showed that shDNMT1 infection blocked the EZH2-induced reduction in cellular cholesterol efflux. Interestingly, shDNMT1 infection alone significantly increased cellular cholesterol efflux, suggesting high endogenous activities of DNMT1. Furthermore, shDNMT1 infection in RAW264.7 macrophage-derived foam cells prevented EZH2-induced cholesterol accumulation. Notably, shDNMT1 infection alone prevented foam cell formation (Fig 5F). Thus, the effects of EZH2 on cell cholesterol efflux and lipid accumulation might result from DNA methylation of ABCA1 promoter.

Finally, HPLC was conducted to determine cellular cholesterol contents. The concentrations of total cellular cholesterol, free cholesterol and cholesterol ester in EZH2-overexpressed cells were significantly higher than those in the control cells, especially when cells were infected with LV-EZH2 vector for 24 h (Tables 2 and 3). When 5-Aza-dC or shDNMT1 was used to inhibit DNMT activity, EZH2 failed to increase cellular cholesterol levels. EZH2 overexpression obviously decreased plasma HDL-cholesterol levels and slightly, but non-significantly, increased LDL-cholesterol levels *in vivo*. EZH2 overexpression also slightly, but not significantly, decreased plasma total cholesterol levels (Table 4). Taken together, these findings indicate that EZH2 decreases cholesterol efflux, and increases cholesterol levels in foam cells likely via DNA methylation and silencing of ABCA1 gene.

**Table 2:** EZH2 increased cholesterol levels in THP-1-derived foam cells via DNA methylation.

	control	5-Aza-dC	EZH2	5-Aza-dC+EZH2
TC (mg/g protein)	503±28	391±32*	594±37^#^	508±29
FC (mg/g protein)	190±27	138±31*	242±24^#^	191±33
CE (mg/g protein)	314±32	174±26*	454±35^#^	312±32
CE/TC (%)	61.9	44.5	76.4	61.4

TC: total cholesterol, FC: free cholesterol, CE: cholesterol ester.

*: *P<*0.05 *vs*. control

#: *P<*0.05 *vs*. 5-Aza-dC+EZH2.

**Table 3:** EZH2 increased cholesterol levels in RAW264.7 macrophage-derived foam cells via DNA methylation.

	control	5-Aza-dC	EZH2	5-Aza-dC+EZH2
TC (mg/g protein)	506±26	384±37*	587±32^#^	509±28
FC (mg/g protein)	193±31	126±33*	251±24^#^	195±33
CE (mg/g protein)	309±30	168±31*	449±33^#^	314±25
CE/TC (%)	61.1	43.8	76.5	61.7

TC: total cholesterol, FC: free cholesterol, CE: cholesterol ester.

*: *P<*0.05 *vs*. control

#: *P<*0.05 *vs*. 5-Aza-dC+EZH2.

**Table 4:** Body weight and plasma lipid profile in apoE^−/−^ mice.

	Body weight(g)	TG(mmol/l)	TC(mmol/l)	HDL-C(mmol/l)	LDL-C(mmol/l)
control	28.55±3.31	0.85±0.10	11.39±1.77	1.52±0.19	9.84±1.16
HF	30.06±4.67	1.05±0.14*	21.10±2.46*	1.98±0.27*	19.09±2.65*
HF+EZH2	29.36±3.98	1.07±0.15*	20.84±2.38*	1.55±0.21^#^	19.27±2.43*

Plasma samples from different experimental groups were measured by the enzymic method. The data were expressed as mean ± S.D. from all the male apoE^−/−^ mice in each group. TC, total cholesterol; TG, Triglyceride; HDL-C, high density lipoprotein-cholesterol; LDL-C, low density lipoprotein- cholesterol.

*: *P*<0.05 *vs*. control group

#: *P*<0.05 *vs*. HF group.

## Discussion

**Fig 6:**
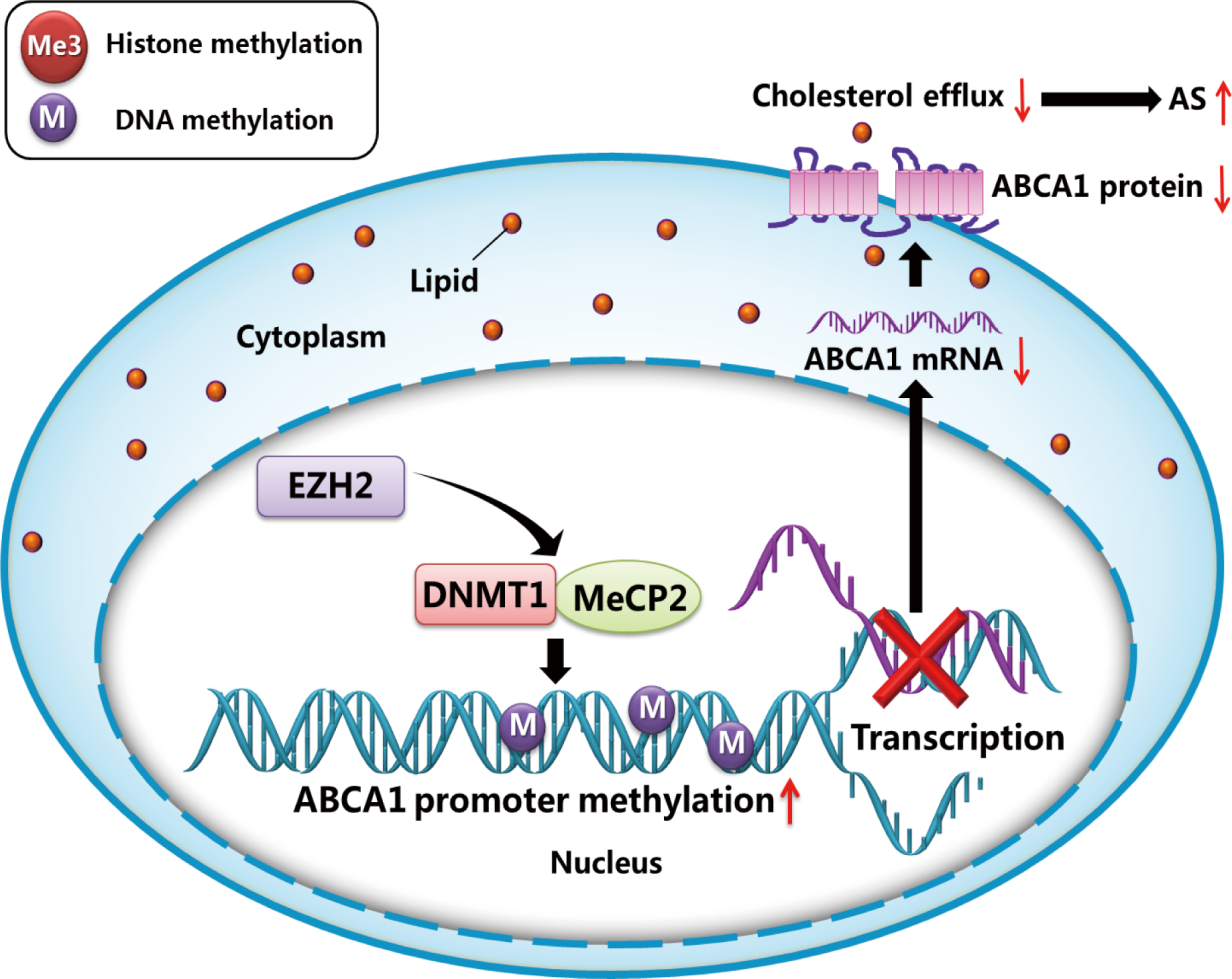
Schematic of proposed mechanism by which EZH2 downregulates macrophage ABCA1 expression leading to impaired cholesterol efflux. EZH2 recruits DNMT1 and MeCP2 to the promoter of ABCA1 gene, which mediated the CpG methylation of ABCA1 promoter, and causing the transcriptional silencing of macrophage ABCA1 expression and subsequent impairment of cholesterol efflux.

Epigenetic regulation plays important roles in a wide range of biological processes, such as gene silencing [18, 19]. However, the epigenetic mechanisms involved in the regulation of ABCA1 gene expression are not yet fully clarified. Here, we reported that the histone methyltransferase EZH2 regulates the expression of DNMT1 and subsequently promotes DNA methylation of ABCA1 promoter, resulting in silencing of ABCA1 gene (Fig 6). These findings have provided further insights into the complexity of the epigenetic regulatory effects on atherosclerosis development.

ABCA1 expression can be regulated by a wide range of factors, such as ox-LDL, liver X receptor/retinoid X receptor, microRNAs, inflammation, and various transcriptional activators and repressors. The ABCA1 promoter region is highly enriched with CpGs, and contains well-defined CGIs. Many CpG dinucleotides in this island are conserved among different species. All these information reinforces the concept that CpG hypermethylation may contribute to transcriptional regulation of ABCA1 gene expression. Our data suggest that EZH2 induces the recruitment and binding of DNMT1 and MeCP2 to the ABCA1 promoter, leading to ABCA1 silencing and acceleration of foam cell formation.

EZH2 is a core member of polycomb repressive complex-2 that mediates repressive H3 histone K27 lysine trimethyltransferase activity of the chromatin [20]. In addition to histone methyltransferase activity, EZH2 has been reported to directly control DNA methylation through its association with and regulation of the activity of DNA methyltransferases [21]. Our results have revealed that EZH2 overexpression induced ABCA1 DNA methylation, suggesting that EZH2-induced hypermethylation and corresponding recruitment of the DNA methylation machinery are within the proximal region of ABCA1 promoter, and may be sufficient to silence the ABCA1 gene.

It is well recognized that the changes in CpG methylation occur during the pathogenesis of atherosclerosis [22–25]. However, whether CpG hypermethylation or hypomethylation plays a causative role in atherogenesis remains controversial. Newman suggested that reduced global DNA methylation increases the progression of atherosclerosis, which is linked to the inadequate production of S-adenosylmethionine in serum [26]. Similarly, in a hypercholesterolemic mouse model, atherogenic lipoproteins result in an aberrant DNA methylation pattern [27]. A more recent study showed a novel functional role for a 3’-exon CGI, supporting a modified mechanism for the roles of apoE as a disease risk [28]. These studies provide support for the role of CpG hypomethylation of specific genes in relevant cardiovascular cell types in promoting atherogenesis. These studies also support the involvement of EZH2 in DNMT1 recruitment and reveal a novel role for EZH2 in cardiovascular disease [29]. In contrast, some reports have shown hypermethylation of estrogen receptor-α and β genes in human atherosclerotic lesions [30]. Therefore, both hypermethylation and hypomethylation of targeted genes may contribute to the development of atherosclerotic disease.

Among the mammalian DNMTs, DNMT1 maintains CpG methylation in postnatal tissues, whereas DNMT3s (DNMT3a, DNMT3b and DNMT3L) mediate de novo methylation and are the principal methyltransferase that contributes to a wave of methylation that occurs during embryogenesis [31]. DNA methylation represses gene expression partially by recruiting MeCP2 that selectively interacts with methylated CpG dinucleotides [32]. Thus, MeCP2 may repress the gene expression by chromatin condensation independent of histone acetylation [33]. Therefore, MeCP2 may induce DNA methylation to alter gene expression. Our findings indicate that the repression of ABCA1 expression by EZH2 is mediated by MeCP2, consistent with the role of EZH2 in silencing the expression of some anticancer genes in tumor cells [34].

Although our studies were limited to macrophages, ABCA1 is expressed in circulating leukocytes and liver cells. Monocytes are transformed to macrophages with a vital role in foam cell formation and vascular inflammation associated with atherosclerosis. Notably, the expression of ABCA1 in peripheral blood monocytes inversely correlates with the extent of atherosclerotic disease [15], suggesting that epigenetic silencing of ABCA1 gene expression in monocytes and macrophages may contribute to atherosclerosis. In the present study, we observed that EZH2 downregulated ABCA1 expression in macrophage-derived foam cells, which exacerbate the formation of atherosclerotic plaque in artery wall. Our data also suggest a molecular mechanism by which EZH2-induced downregulation of ABCA1 involves upregulation of DNMT1 and MeCP2 activities and enhanced recruitment of these factors to ABCA1 promoter region. It must be noted that this epigenetic mechanism was also implicated in the downregulation of hepatic ABCA1 in apoE^−/−^ mice with EZH2 overexpression, which accordingly impaired the lipidation of hepatic apoA-I and the recycling apoA-I at the hepatocyte surface and accelerated aortic atherosclerosis development [35]. Furthermore, it is possible that EZH2 silenced ABCA1 expression via other epigenetic mechanisms such as histone modification [36], but whether these mechanisms participate in ABCA1 expression needs to be explored in the future studies.

In conclusion, our findings obtained from apoE^−/−^ mice provide epigenetic insights into how EZH2 increases the risk of atherosclerotic heart disease. One of the pathways by which EZH2 leads to lipid accumulation and foam cell formation is via epigenetic downregulation of ABCA1 expression. Therefore, targeted inhibition of ABCA1 DNA methylation, if achievable, could provide a new therapeutic approach to combating atherosclerosis and cardiovascular diseases.

## Supporting Information

S1 Fig A, EZH2 protein expression was increased in THP-1 cells, RAW264.7 macrophage and MPM in response to infection with LV-mock or LV-EZH2 for 24 h. B, EZH2 protein expression was increased in aorta, heart, liver and kidney of apoE^−/−^ mice infected with LV-mock or LV-EZH2. Mean ± S.D., *: *P*<0.05 *vs*. control, experiments were performed in triplicate.
(TIF)

S2 Fig DNMT1 protein expression was determined in THP-1 and RAW264.7 macrophages infected with LV-mock, LV-DNMT1 or LV-shDNMT1 for 24 h. Mean ± S.D., *: *P*<0.05 *vs*. control, experiments were performed in triplicate.
(TIF)

S3 Fig MeCP2 protein expression was increased in THP-1 and RAW264.7 macrophages infected with LV-mock or LV-MeCP2 for 24 h. Mean ± S.D., *: *P*<0.05 *vs*. control, experiments were performed in triplicate.
(TIF)
