# Supplementary figures and images for: Histone Methyltransferase Enhancer of Zeste Homolog 2-Mediated ABCA1 Promoter DNA Methylation Contributes to the Progression of Atherosclerosis
Source: PLoS One. 2016 Jun 13;11(6):e0157265. doi: 10.1371/journal.pone.0157265 (PMC4905646; doi:10.1371/journal.pone.0157265)

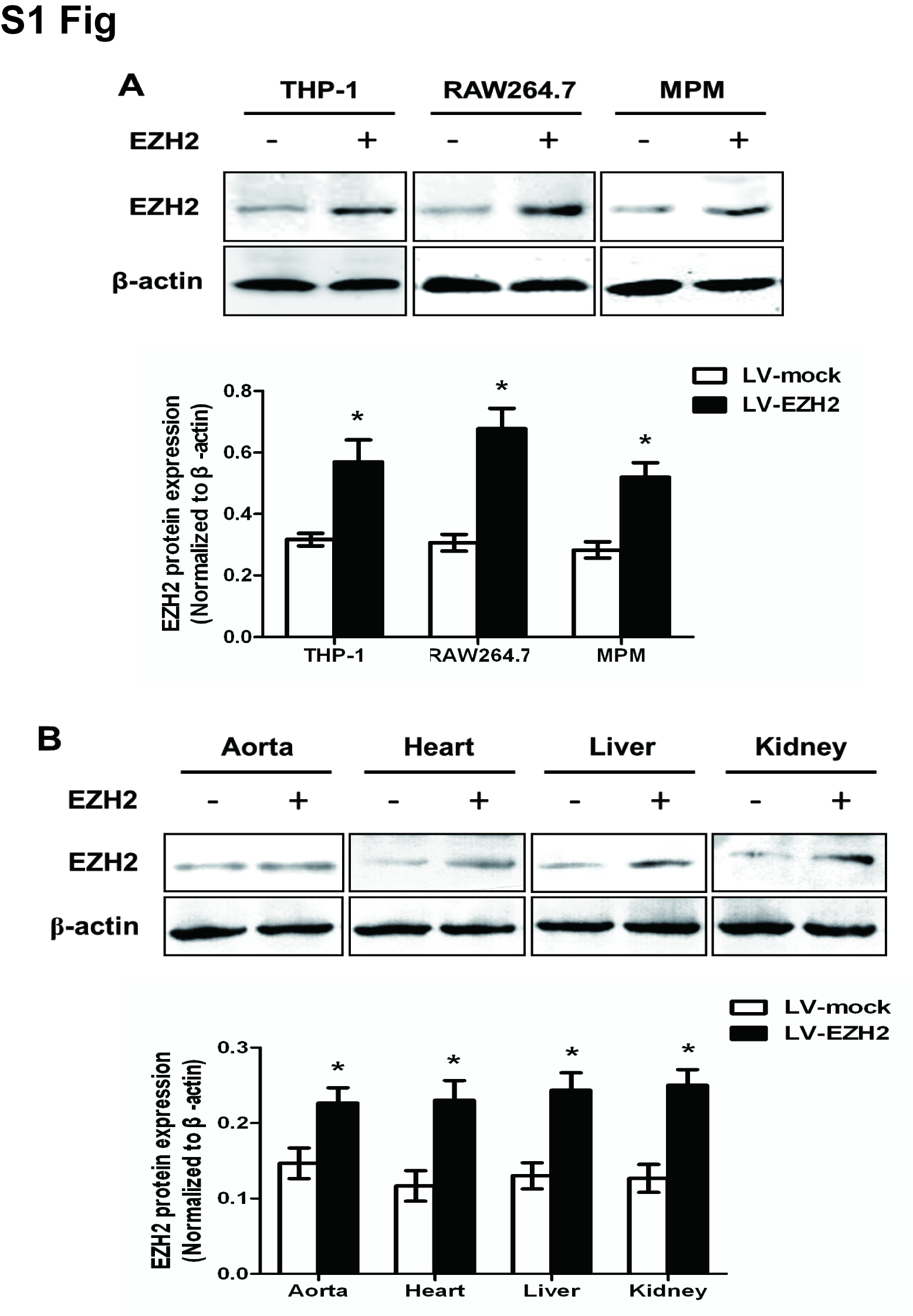

Supplement: S1 Fig — A, EZH2 protein expression was increased in THP-1 cells, RAW264.7 macrophage and MPM in response to infection with LV-mock or LV-EZH2 for 24 h. B, EZH2 protein expression was increased in aorta, heart, liver and kidney of apoE−/− mice infected with LV-mock or LV-EZH2. Mean ± S.D., *: P<0.05 vs. control, experiments were performed in triplicate. (TIF) [file pone.0157265.s001.tif]

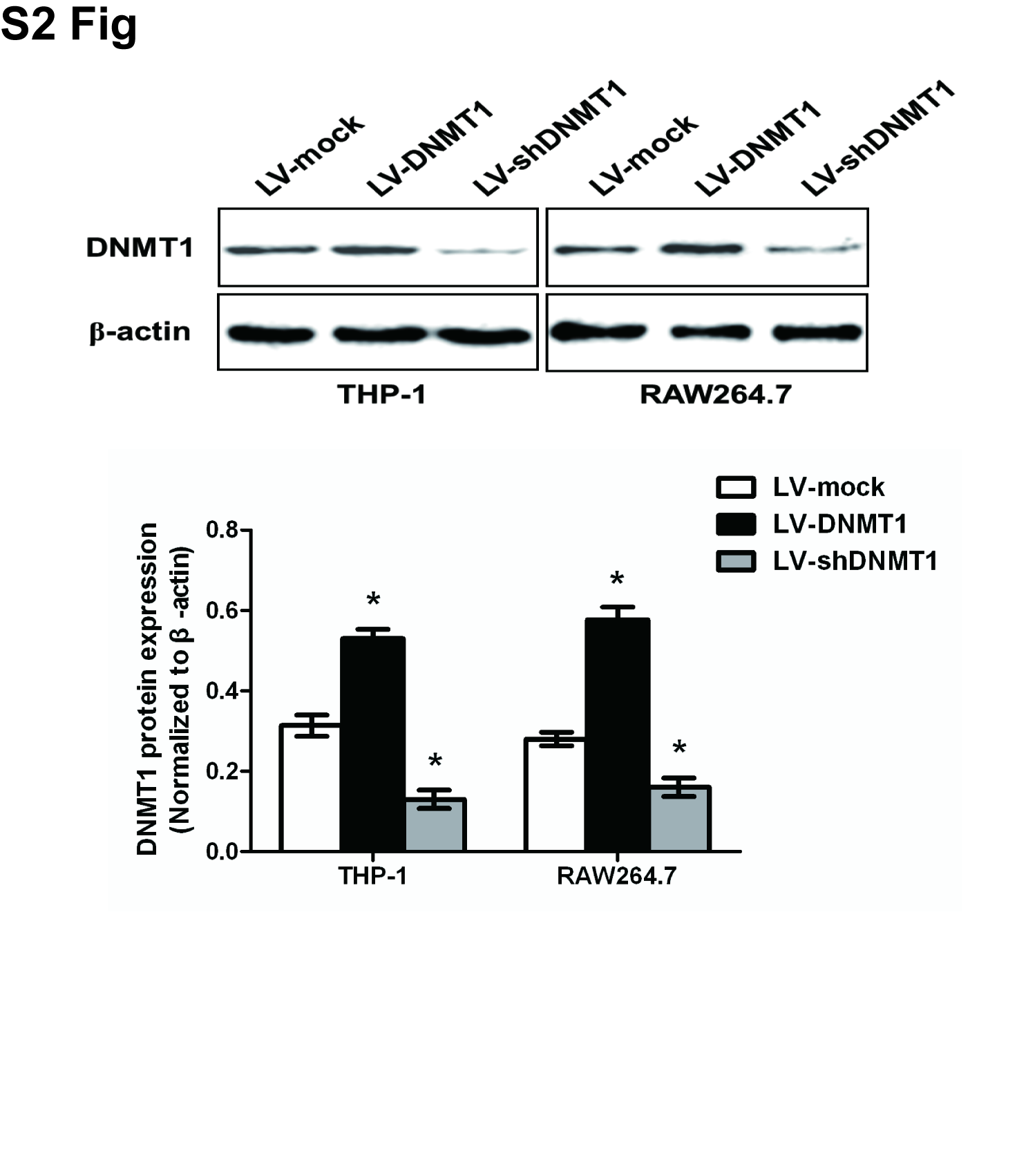

Supplement: S2 Fig — Mean ± S.D., *: P<0.05 vs. control, experiments were performed in triplicate. (TIF) [file pone.0157265.s002.tif]

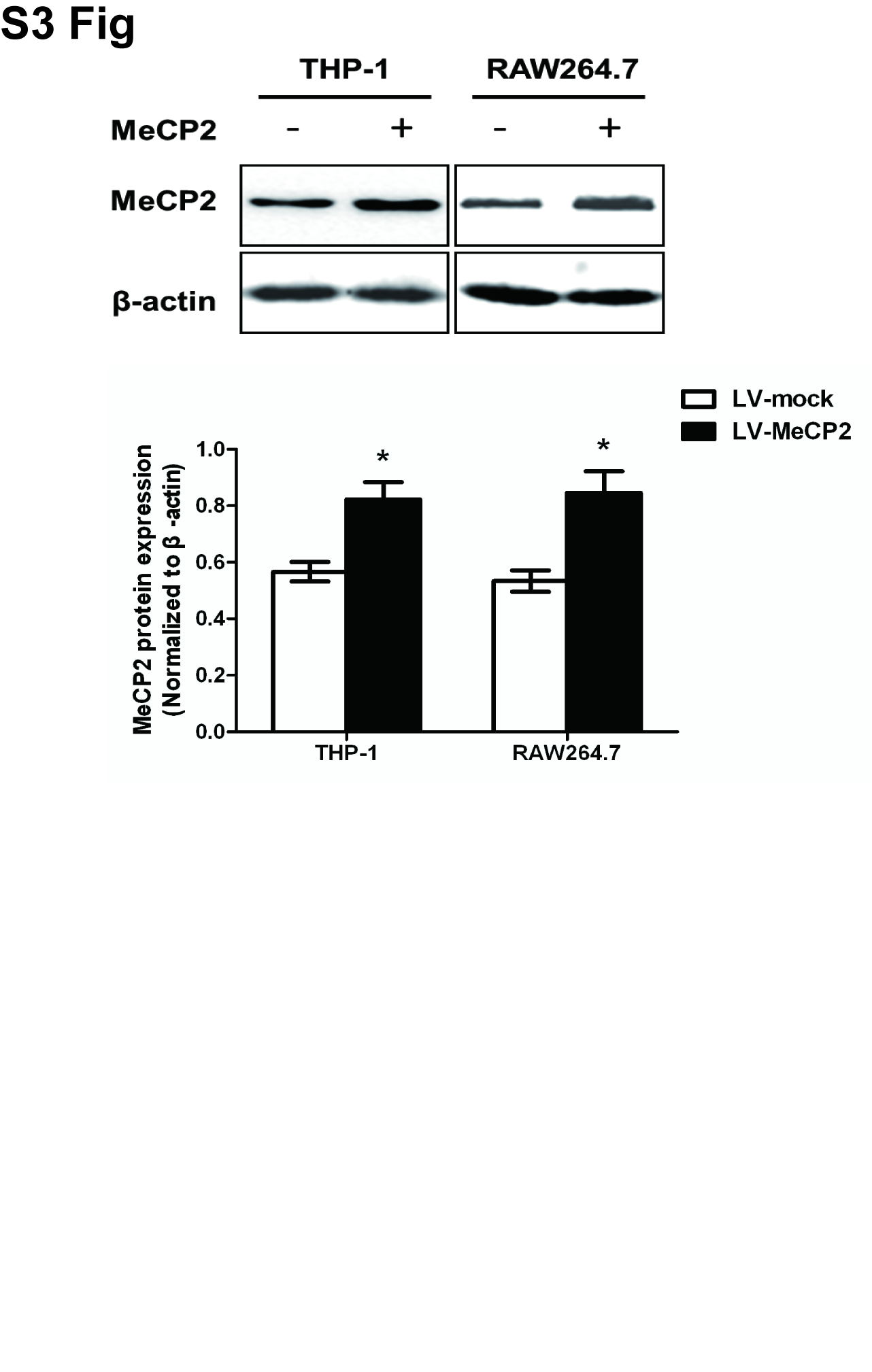

Supplement: S3 Fig — Mean ± S.D., *: P<0.05 vs. control, experiments were performed in triplicate. (TIF) [file pone.0157265.s003.tif]
